# Post-ictal Psychosis: clinical case presentation and literature review

**DOI:** 10.1192/j.eurpsy.2025.1249

**Published:** 2025-08-26

**Authors:** F. Ramalheira, M. D. C. Vasconcelos, M. Andrade, M. Pereira

**Affiliations:** 1 Hospital Júlio de Matos, Unidade Local de Saúde de São José, Lisboa, Portugal

## Abstract

**Introduction:**

Post-ictal psychosis is a kind of epileptic psychosis in which psychotic features appear 12h – 7 days after the epileptic seizure causing them.

**Objectives:**

To present a challenging case reflecting upon presentation, management and considerations of post-ictal psychosis.

**Methods:**

Case presentation and non-systematic literature review.

**Results:**

A 40-year-old female, living in a European foreign country with her partner and child, flies to Lisbon 5 days previously to her admission as a sudden decision because she believed she was the daughter of a portuguese yoga master. She was brought to psychiatry ER by the police due to disorganized and aggressive behavior in public, where she presented with severe agitation, disorganized and coprolalic speech, persecutory, mistic and filiation delusions, somatic and affective passivity, and very uncollaborative. She had no analytic or image alterations except for positive cannabinoids in urine. She was admitted in psychiatry and started on risperidone titled till 6mg and diazepam 15mg, with remission of symptomatology. When she was able to collaborate, she admitted she had a history of Epilepsy for which she was not having treatment, and a previous post-ictal psychotic episode some years ago. Family confirmed she had a generalized tonic-clonic seizure about a week ago, and delusional ideas starting the following day, having left home unannounced. Although the EEG was normal, considering suggestive history post-ictal psychosis was admitted ad most probable diagnosis and she was slowly stopped medication without symptom resurgence. The importance of anti-epileptic treatment in order to avoid subsequent seizures and post-ictal psychosis was explained, however the patient denied treatment and was discharged back to her hometown with indication to follow-up in neurology.

**Conclusions:**

Post-ictal psychosis corresponds to 25% of epileptic psychosis. It is associated with temporal lobe epilepsy, psychotic symptoms of mistic and religious themes, aggressive behavior, and increased suicide risk. It has a sudden onset and complete remission although a risk of recurrence as in this case. Treatment consists of seizure control to avoid following episodes. Antipsychotics that don’t increase convulsive risk such has risperidone may be useful to acute control of psychotic features and behavior alterations.

**Disclosure of Interest:**

None Declared

